# Diabetes, perioperative ischaemia and volatile anaesthetics: consequences of derangements in myocardial substrate metabolism

**DOI:** 10.1186/1475-2840-12-42

**Published:** 2013-03-04

**Authors:** Charissa E van den Brom, Carolien SE Bulte, Stephan A Loer, R Arthur Bouwman, Christa Boer

**Affiliations:** 1 Department of Anesthesiology, Experimental Laboratory for VItal Signs (ELVIS), VU University Medical Center, De Boelelaan 1117, Amsterdam, 1081 HV, the Netherlands; 2 Laboratory for Physiology, VU University Medical Center, Van der Boechorststraat 7, Amsterdam, 1081 BT, the Netherlands

**Keywords:** Volatile anaesthetics, Substrate metabolism, Ischaemia, Diabetes, Heart

## Abstract

Volatile anaesthetics exert protective effects on the heart against perioperative ischaemic injury. However, there is growing evidence that these cardioprotective properties are reduced in case of type 2 diabetes mellitus. A strong predictor of postoperative cardiac function is myocardial substrate metabolism. In the type 2 diabetic heart, substrate metabolism is shifted from glucose utilisation to fatty acid oxidation, resulting in metabolic inflexibility and cardiac dysfunction. The ischaemic heart also loses its metabolic flexibility and can switch to glucose or fatty acid oxidation as its preferential state, which may deteriorate cardiac function even further in case of type 2 diabetes mellitus.

Recent experimental studies suggest that the cardioprotective properties of volatile anaesthetics partly rely on changing myocardial substrate metabolism. Interventions that target at restoration of metabolic derangements, like lifestyle and pharmacological interventions, may therefore be an interesting candidate to reduce perioperative complications. This review will focus on the current knowledge regarding myocardial substrate metabolism during volatile anaesthesia in the obese and type 2 diabetic heart during perioperative ischaemia.

## Introduction

Perioperative cardiac complications occur in 2-5% of all non-cardiac surgical procedures, which globally affect 5–12 million patients each year [1]. More specifically, 0.65% of these patients develop perioperative myocardial infarction or cardiac arrest [2]. Perioperative cardiac complications are an economical, medical and social burden that warrants optimisation of perioperative health and cardiovascular care to improve patient outcome and reduce health care costs. There are several well-known predictors for perioperative cardiac complications identified, such as type of surgery, ASA classification and increasing age [1,2]. Additionally, lifestyle risk factors associated with metabolic alterations, such as excessive dietary intake and physical inactivity, are strongly associated with clinical risk factors that predict perioperative cardiovascular complications [1].

Lifestyle risk factors related to obesity and type 2 diabetes mellitus (T2DM) have become an epidemic over the last decade. Worldwide, 366 million people have T2DM [3]. It is predicted that in the year 2030 about 552 million people will have overt diabetes, mainly T2DM [3]. Patients with T2DM are more likely to develop coronary artery disease and myocardial ischaemia [4] and have an increased cardiovascular complication rate after major non-cardiac surgery [5].

In addition to prevention programs to reduce the burden of metabolic disease on the perioperative process, there are intraoperative cardioprotective strategies available that may reduce the impact of ischaemic injury during and after surgery, like the application of the volatile anaesthetics sevoflurane and isoflurane. These volatile anaesthetics exert multiple protective effects that enhance perioperative preservation of the heart in patients [6] and rats [7]. Although exposure to volatile anaesthetics reduced infarct size and improved post-ischaemic recovery in healthy rats [7], the cardioprotective effects of these agents are reduced in obese [8] and hyperglycaemic [9] rats. Derangements in myocardial substrate metabolism are one of the hypothetical mechanisms that may explain the suppressed cardioprotective capacity in T2DM [10-12]. It is however not yet understood how these myocardial metabolic alterations affect intraoperative cardioprotective mechanisms.

In order to elucidate the impact of altered myocardial substrate metabolism on intraoperative myocardial protection, this review will focus on available preclinical knowledge regarding myocardial substrate metabolism during volatile anaesthesia in the obese/T2DM heart under normal conditions and in the context of ischaemia. We first describe myocardial substrate metabolism under healthy, obese/T2DM and ischaemic conditions, followed by an overview of the interaction between substrate metabolism and volatile anaesthetics in the context of perioperative ischaemia and reperfusion injury. Finally, we propose strategies to modulate myocardial substrate metabolism that may contribute to an improvement of myocardial protective capacity and perioperative and postoperative outcome in obesity and T2DM.

## Myocardial substrate metabolism

Fatty acids and carbohydrates are essential for the pump function of the heart [13]. Under physiological conditions, myocardial contractile function relies on oxidation of fatty acids (60-70%), glucose (30-40%) and to a lesser extent lactate, ketones, amino acids and pyruvate (10%) to generate adenosine triphosphate (ATP) [14-16]. The heart exerts a metabolic flexibility, and myocardial substrate utilisation depends on substrate availability, nutritional status, and exercise level. With glucose as the more energetically efficient substrate, the healthy heart is able to switch to glucose under conditions of stress, such as ischaemia, pressure overload or in heart failure.

**Figure 1:**
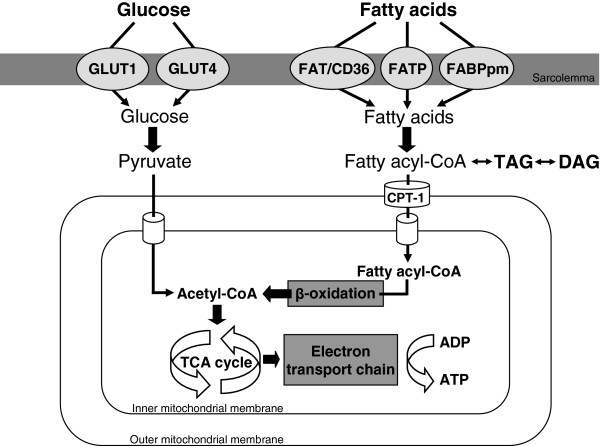
**Glucose and fatty acid metabolism in the cardiomyocyte.** Glucose uptake into the cell occurs through the glucose transporters GLUT1 and GLUT4. Once inside, glucose is broken down into pyruvate by glycolysis. Pyruvate is subsequently transported into the mitochondria and decarboxylated to acetyl-CoA. Non-esterified fatty acids are taken up through fatty acid transporter (FAT)/CD36, fatty acid transport protein (FATP) and plasma membrane fatty acid binding protein (FABPpm). Intracellular fatty acids form fatty acyl-CoA and can either be esterified into triglycerides (TG) or enter the mitochondria via carnitine palmitoyl transferase (CPT-1). Fatty acyl-CoA enters the β-oxidation pathway, forming acetyl-CoA. Glucose or fatty acid-derived acetyl-CoA enters the tricarboxylic acid (TCA) cycle with entry of reducing equivalents to the electron transport chain and oxidative phosphorylation, and finally ATP is formed.

Glucose metabolism is regulated through multiple steps, including uptake, glycolysis and pyruvate decarboxylation. Myocardial glucose supply is regulated 1) via circulating glucose levels or 2) by release of glucose from intracellular glycogen stores [17]. Myocardial glucose uptake depends on the sarcolemmal glucose transporter GLUT1 (insulin-independent) and the dominant glucose transporter GLUT4 (insulin-dependent) (Figure 1) [18]. After uptake, glucose is broken down into pyruvate by glycolysis, consumed by the mitochondria and decarboxylated into acetyl-CoA by pyruvate dehydrogenase. Acetyl-CoA enters the tricarboxylic acid cycle with entry of reducing equivalents to the electron transport chain and oxidative phosphorylation, which finally leads to ATP formation (Figure 1).

Fatty acid metabolism consists of uptake, oxidation and esterification. There are two sources of fatty acids for myocardial metabolism: 1) circulating albumin bound fatty acids derived from adipose tissue via lipolysis or 2) released from triglyceride-rich lipoproteins from the liver [19]. Fatty acids enter cardiomyocytes by simple diffusion and via transport through three different membrane fatty acid transporters – fatty acid translocase (FAT)/CD36, fatty acid transport protein (FATP1/6) and plasma membrane fatty acid binding protein (FABPpm) (Figure 1) [19]. After sarcolemmal uptake, intracellular fatty acids are activated to form fatty acyl-CoA, which can undergo beta-oxidation or esterification to form intracellular triglycerides [20]. Fatty acid oxidation requires fatty acyl-CoA entry into the mitochondria, which is dependent on the activity of carnitine palmitoyl transferase (CPT-1) [21]. After translocation into the mitochondria, fatty acyl-CoA can enter the beta-oxidation pathway to form acetyl-CoA and subsequently ATP (Figure 1). Under physiological conditions, 70-90% of the fatty acids that enter cardiomyocytes are oxidised for ATP generation, whereas 10-30% is converted to triglycerides by lipoprotein lipase [22]. In case of energy expenditure, intracellular triglyceride stores can be hydrolysed as an endogenous fatty acid source, which is explanatory for 10% of the total fatty acid utilisation in the heart [23].

### Type 2 diabetes mellitus

Alterations in myocardial substrate metabolism in T2DM hearts are extensively reviewed by others [15,22,24]. In short, myocardial fatty acid metabolism is initially enhanced in T2DM hearts, with increased rates of fatty acid oxidation and esterification [25,26]. There are two proposed mechanisms that may underlie this derangement: 1) increased fatty acid uptake due to increased substrate supply and augmented expression and localisation of sarcolemmal fatty acid transporters [26] and 2) increased oxidation and esterification due to changes in regulation at both the enzymatic and transcriptional level [26].

In addition, a decreased myocardial glucose metabolism is a concomitant feature of the T2DM heart [25,26]. The slow rate of glucose transport across the sarcolemmal membrane due to decreased glucose transporters leads to a restriction of glucose oxidation. Accordingly, fatty acid oxidation has an inhibitory effect on the pyruvate dehydrogenase complex due to increased fatty acid supply. Taken together, the T2DM heart has a distinct metabolic phenotype, characterised by enhanced myocardial fatty acid metabolism and a concomitant reduction in myocardial glucose metabolism.

### Ischaemia

Myocardial ischaemia occurs when coronary perfusion is inadequate to maintain a sufficient oxygen supply/demand ratio. Ischaemia influences both myocardial substrate metabolism and myocardial function. The pathophysiological mechanisms underlying this phenomenon have been reviewed previously [24,27].

In the event of ischaemia, high-energy phosphates are depleted, ionic homeostasis is disturbed and contractile dysfunction is caused. The energetic demand of the heart changes in case of myocardial ischaemia. The heart usually responds to injury by increasing myocardial glucose metabolism to improve its energetic efficiency [22,24]. However, increased adipose tissue lipolysis results in increased plasma free fatty acid concentrations, which may increase myocardial fatty acid utilisation and esterification [27]. In this context, glycolysis becomes an important source of energy due to its ATP-generating ability in the absence of oxygen. It is also suggested that in the early phase of ischaemia, fatty acid oxidation shifts to the more efficient glucose oxidation, followed by a decrease in total substrate oxidation [24]. Increased glycolysis can parallel depression of myocardial glucose and fatty acid oxidation depending on the severity of ischaemia. Overall, the ischaemic heart favours the energetically more efficient glucose (3.17 ATP/oxygen molecule) over fatty acid oxidation (2.83 ATP/oxygen molecule) [28]. This flexibility additionally depends on substrate availability, oxygen supply, tissue vascularisation and myocardial workload. In conclusion, the metabolic state of the ischaemic heart is characterised by imbalances in substrate availability and utilisation and is also influenced the severity of ischaemia.

### The combination of type 2 diabetes mellitus and ischaemia

The cardiometabolic profile of patients with T2DM makes them more prone to develop plaque formation and intravascular stenosis, leading to the development of stroke or myocardial infarction. In addition, these patients are more susceptible to subsequent episodes of ischaemia [29,30]. Whereas the metabolic undisturbed heart usually responds to injury by increasing myocardial glucose metabolism [22,24], this adaptive response is inhibited by insulin resistance, which is a characteristic of obesity and T2DM. This inhibition results in increased myocardial fatty acid metabolism [31,32], increased oxygen consumption, decreased cardiac efficiency [31] and altered myocardial perfusion [33]. In obese or T2DM animals subjected to myocardial ischaemia the findings are inconclusive. It has been shown that obesity reduced ischaemia and reperfusion injury [34] and myocardial function during ischaemia (and reperfusion) [35-40], but also similar ischaemia and reperfusion injury was found [41]. Additionally, increased glucose oxidation and decreased fatty acid oxidation after myocardial infarction was found, which was ameliorated in obese rats [40]. Obese rats with insulin resistance resulted in preserved myocardial function [36] or aggravated [36,42-44] ischaemia and reperfusion injury. Moreover, the combination of insulin resistance, dyslipidaemia and hypertension in obese animals seems to increase the susceptibility of the heart to ischaemia (and reperfusion) injury [45-48]. Others however reported that myocardial injury during ischaemia was unaffected in T2DM rats, independent of the severity of T2DM [49]. In case of genetically induced T2DM rats in combination with a high cholesterol diet, ischaemic injury was however exacerbated [50]. As stated earlier, these inconclusive results in animal experiments suggest that the type and severity of T2DM may influence the sensitivity of the heart to ischaemic insults.

With regard to myocardial substrate metabolism, endogenous glycogen stores may support increased glucose availability as substrate for the heart, and may thus be beneficial in case of ischaemic injury. However, whether pre-ischaemic glycogen levels are beneficial or detrimental depends on the duration of T2DM [51] and to the extent of glycogen depletion during ischaemia [52].

Overall, the effects of imbalanced myocardial substrate metabolism during ischaemia in T2DM are inconclusive. These observed contrasts may be due to differences in the severity of ischaemia, the measured outcome parameter, exogenous circumstances and the severity of the experimental model for T2DM [32,53].

## Effects of volatile anaesthetics in animals

### Cardioprotective effects during ischaemia

Sevoflurane and isoflurane are commonly used volatile anaesthetics. Sevoflurane and isoflurane make the rat heart more resistant to ischaemia and reperfusion injury [54-58]. It has been shown that proteins related to myocardial substrate metabolism are, amongst others, affected by sevoflurane-induced cardioprotection. PI3K and Akt, which regulate translocation of glucose transporter 4 (GLUT4) to the sarcolemma for glucose uptake, are increased during sevoflurane in the isolated ischaemic rat heart [59]. Moreover, sevoflurane enhances GLUT4 expression in lipid rafts, increases glucose oxidation and decreases fatty acid oxidation after ischaemia and reperfusion injury in isolated working rat hearts compared to untreated ischaemic hearts [10]. In the same study, no alterations in AMP activated protein kinase (AMPK) phosphorylation, pyruvate dehydrogenase activity and glycogen content were found, whereas sevoflurane decreased triglycerides and ceramide levels after ischaemia and reperfusion injury [10].

Moreover, volatile anaesthetics are also known to alter mitochondrial function, which is nicely reviewed by Stadnicka *et al.*[60]. In short, it has been shown that sevoflurane and isoflurane open mitochondrial ATP-activated potassium (mito K_ATP_) channels [61,62], activates reactive oxygen species [62] and thereby alters mitochondrial metabolism [63].

Together, these results suggest a role for myocardial substrate metabolism in the cardioprotective effects of volatile anaesthesia during ischaemia and reperfusion injury in animals, although evidence is limited.

### Myocardial substrate metabolism during volatile anaesthesia

In rats, it has been shown that *in vivo* myocardial glucose uptake was increased in the heart during isoflurane (2 vol%) when compared to sevoflurane (3.5 vol%) [64]. An explanation could be the differences by more stable blood glucose levels during sevoflurane. However, a limitation of this study was that the effects were not compared with findings in awake rats or using non-volatile anaesthetics. Others found that isoflurane (2 vol%) increased myocardial glucose uptake compared to awake mice [65].

The effects of sevoflurane on myocardial substrate metabolism have only been studied *ex vivo*. Sevoflurane (2 vol%) decreased FAT/CD36 in lipid rafts and fatty acid oxidation in isolated rat hearts [12]. And, although studied in skeletal muscle cells, sevoflurane (2.6-5.2%) increased glucose uptake [66]. Altogether, these results suggest that isoflurane and sevoflurane might switch myocardial metabolism to glucose as energetically more efficient substrate.

Volatile anaesthesia is also known to affect pancreatic insulin release. In isolated rat pancreatic islets, enflurane [67] and isoflurane [68] have an inhibitory effect on glucose-stimulated insulin release. In rats, isoflurane impaired glucose-induced insulin release [69], whereas sevoflurane impaired glucose tolerance [70], which both resulted in hyperglycaemia. Therefore it seems that impaired insulin release during volatile anaesthesia might have a negative effect on substrate metabolism. However, the beneficial cardioprotective effects may outweigh the adverse effects of impaired insulin secretion, as the American Heart Association 2007 guidelines on ‘perioperative cardiovascular evaluation and care for non cardiac surgery’ suggested that it can be beneficial to use volatile anaesthetics during non cardiac surgery for maintenance of general anaesthesia in haemodynamically stable patients at risk for myocardial ischaemia [1].

### Alterations in cardioprotective mechanisms in the metabolic altered heart

The healthy heart is capable of protecting itself against stressors like ischaemia by the flexibility to switch between circulating substrates. These cardioprotective properties might be enlarged during volatile anaesthesia. On the other hand, the obese/T2DM heart is less capable of switching between circulating substrates, which may contribute to a reduced intrinsic protective capacity. It is generally acknowledged that the incidence of perioperative cardiovascular complications is increased in patients with T2DM after non-cardiac surgery [5]. Accordingly, blood glucose concentrations at admission correlated with long-term mortality in diabetic patients with acute myocardial infarction [71], suggesting that T2DM may affect perioperative cardiovascular risk. The next paragraphs focus on available experimental knowledge whether obesity, insulin resistance, hyperlipidaemia and hyperglycaemia, important hallmarks of T2DM, exert a cumulative effect on endogenous and exogenous cardioprotective mechanisms.

#### Obesity and insulin resistance

It has been shown that obesity and insulin resistance inhibit the cardioprotective effects of ischaemic pre- [72] and postconditioning [73]. In high fat diet-induced obese rats, sevoflurane preconditioning failed to induce cardioprotection during myocardial ischemia and reperfusion injury [41]. Moreover, sevoflurane postconditioning did not protect the heart against myocardial and reperfusion injury in obese and insulin resistant Zucker rats [8], however, more research is necessary to draw a conclusion.

#### Hyperlipidaemia

The hyperlipidaemic heart has difficulties to adapt to stressors like ischaemia, suggesting that cardioprotective mechanisms are impaired. In rats it has been shown that pacing-induced cardioprotection [74] and ischaemic-induced preconditioning [75] was inhibited by hypercholesterolaemia. Sevoflurane preconditioning reduced myocardial infarct size in normocholesterolaemic rats, which was blocked in hypercholesterolaemic rats [76]. Further research is warranted to study the impact of hyperlipidaemia on anaesthesia-induced cardioprotection.

#### Acute hyperglycaemia

Hyperglycaemia is an independent predictor of cardiovascular risk [71]. The glycometabolic state upon hospital admission is associated with the mortality risk in T2DM patients with acute myocardial infarction [77]. It has further been shown that hyperglycaemia inhibits the cardioprotective capacity during desflurane-induced preconditioning [78], isoflurane-induced preconditioning [9,79] and sevoflurane-induced postconditioning in the experimental setting [80]. Accordingly, infarct size was directly related to the severity of hyperglycaemia [81,82], whereas the inhibited cardioprotective effects of isoflurane-induced preconditioning are concentration dependent and related to the severity of acute hyperglycaemia [9]. Moreover, it has been shown that hyperglycaemia attenuated cardioprotection via inhibition of Akt and endothelial nitric oxide synthase (eNOS) phosphorylation [83]. However, interpretation of abovementioned findings in relation to T2DM is difficult, because experiments were performed during acute hyperglycaemia in otherwise healthy animals without the typical characteristics of T2DM, such as obesity and insulin resistance.

#### Type 2 diabetes mellitus

T2DM hinders the cardioprotective effects of ischaemic preconditioning [84], which has been reviewed by Miki *et al. *[85]. However, the diabetic rat heart may still benefit when the preconditioning stimulus is enlarged [86]. The effects of anaesthesia-induced cardioprotection in T2DM have however never been studied. In type 1 diabetes, the protective effects of isoflurane-induced preconditioning were inhibited in case of low isoflurane concentrations, but not at high concentrations [82]. Further, sevoflurane-induced postconditioning in the type 1 diabetic heart was disturbed, whereas insulin treatment to reach normoglycaemia did not restore the cardioprotective capacity [87]. Mechanisms that are suggested to be involved include the inhibition of PI3K/Akt [86,87] and inactivity of mito K^+^_ATP _[87]. Furthermore, AMPK activation during ischaemia protects the non-obese T2DM Goto-Kakizaki rat heart against reperfusion injury [88], suggesting a role for AMPK in the cardioprotective properties of the diabetic heart. A limitation of the above-described studies is that anaesthesia-induced cardioprotection is only studied in type 1 diabetes with insulinopenia and hyperglycaemia, but without characteristics such as obesity, insulin resistance and hyperinsulinaemia.

Although current findings suggest that the degree of T2DM, dependent on the presence and severity of hyperglycaemia and hyperlipidaemia, is of influence for the cardioprotective capacity of anaesthetics, there are no direct studies available that investigated cardioprotective strategies in animals with this diabetic entity.

## Experimental options to improve perioperative myocardial metabolism

**Figure 2:**
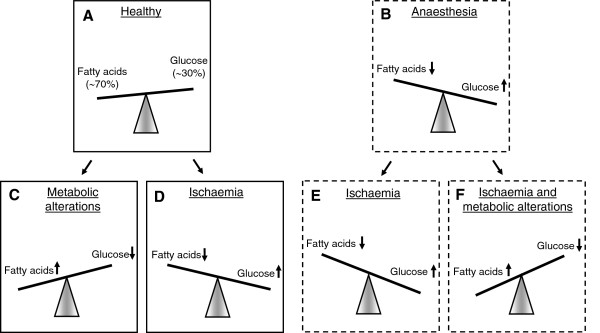
**(Hypothetical) Glucose and fatty acid metabolism under different conditions.** Glucose and fatty acid metabolism in the healthy heart (**A**), during volatile anaesthesia (**B**), in the metabolic altered heart (**C**), in the ischaemic heart (**D**), during volatile anaesthesia in the ischemic heart (**E**) and during volatile anaesthesia in the ischemic metabolic altered heart (**F**). The healthy heart utilises 70% of fatty acids and 30% of glucose for ATP generation (**A**). We hypothesise that one of the mechanisms of volatile anaesthesia is the effect on increased glucose metabolism (**B**). In the metabolic altered heart it is suggested that myocardial substrate metabolism is shifted to increased fatty acid metabolism (**C**), whereas it is suggested that the ischaemic heart is shifted to increased glucose metabolism, however, also contrasting results exist (**D**). We hypothesise that exposure of volatile anaesthetics in the ischaemic heart might increase myocardial glucose metabolism even more (**E**), which is disturbed in the ischaemic and metabolic altered heart (**F**).

The reduced adaptability of the metabolic altered heart to ischaemic injury and cardioprotective interventions warrants further investigation of treatment strategies that optimise myocardial substrate metabolism before surgery. It is suggested that volatile anaesthesia induces a switch from myocardial fatty acid to glucose metabolism. In the metabolically altered heart, however, myocardial substrate metabolism is shifted to increased fatty acid and decreased glucose metabolism. Accordingly, the effect of volatile anaesthetics seems blunted in the metabolic altered heart. As a consequence, an improvement of the metabolic flexibility of the heart may be an important target. Figure 2 shows a hypothetical overview of the effects of different conditions on myocardial substrate metabolism.

### Pharmacological interventions

Improvement of myocardial metabolic flexibility may be achieved by shifting myocardial substrate metabolism to glucose metabolism. This can be induced by 1) altering substrate supply, 2) inhibition of fatty acid oxidation and/or 3) improving insulin sensitivity. The next paragraphs provide an overview of pharmacological interventions in the experimental setting in the treatment of T2DM and/or myocardial ischaemic injury, which might reduce perioperative risk due to normalisation of metabolic derangements (Table 1).

**Table 1:** Overview of pharmacological interventions in the experimental setting

**Drug**	**Applicability**	**Advantages**	**Side-effects**
*Fatty acid metabolism inhibitors*			
Etomoxir	T2DM, infarction [89-91]	Stimulation glucose oxidation [99]	-
Perhexiline	T2DM, infarction [92]	Stimulation glucose oxidation	-
Oxfenicine	T2DM, infarction [92,93]	Stimulation glucose oxidation	-
Trimetazidine	T2DM, infarction [94,95]	Stimulation glucose oxidation	-
Ranolazine	T2DM, infarction [96,97]	Stimulation glucose oxidation [96]	-
Dichloroacetate	T2DM, infarction [98]	Stimulation glucose oxidation [98]	-
*Insulin*			
Glucose-insulin-potassium	Infarction [103]	Stimulation glucose oxidation	Hypoglycaemia
Insulin	T2DM	Reduction glucose levels	Hypoglycaemia
	Infarction [108,109]	Stimulation glucose oxidation	Hypoglycaemia
*PPAR agonists*			
Fibrates (PPARα)	T2DM [47]	Reduction lipids	Myopathy
	Infarction [115-117]	Reduction lipids	Myopathy
Thiozolidinediones (PPARγ)	T2DM [121,122]	Insulin sensitizer	Increased risk heart attacks
	Infarction [44,48,115,119,120]	Insulin sensitizer	Increased risk heart attacks
*Biguanide*			
Metformin	T2DM, infarction [125-128]	Stimulation glucose oxidation	Lactic acidosis
*GLP1*			
GLP1	T2DM, infarction [130]	Reduction glucose	Short half-life
Exendin-4	T2DM, infarction [131]	Reduction glucose	Hypoglycaemia
Exenatide	T2DM, infarction [132]	Reduction glucose	Hypoglycaemia
*Liraglutide*	T2DM, infarction [133,134]	Reduction glucose	Hypoglycaemia

T2DM, type 2 diabetes mellitus; PPAR, peroxisome proliferators-activated receptor; GLP-1, glucagon-like peptide 1.

#### Inhibition of fatty acid metabolism

Carnitine palmitoyl transferase 1 (CPT-1) is a rate-limiting step of fatty acid oxidation. Several inhibitors of CPT-1 have shown beneficial effects during ischaemia and reperfusion in rats, such as etomoxir [89-91], perhexiline [92] and oxfenicine [92,93]. However, not all of these variants of CPT-1 inhibitors are yet registered for clinical use. Other possibilities to reduce fatty acid oxidation are trimetazidine (3-ketoacyl CoA thiolase inhibitor) [94,95], ranolazine (partial fatty acid oxidation inhibitor) [96,97] and dichloroacetate (DCA; pyruvate dehydrogenase kinase inhibitor) [98], which have protective characteristics during myocardial ischaemia in rats. One of the suggested mechanisms underlying the beneficial effects of these substances is the stimulation of myocardial glucose oxidation [96,98,99]. However, as insulin resistance is a hallmark of the metabolic altered heart, stimulation of glucose metabolism via inhibition of fatty acid metabolism may be blunted during insulin resistance. Unfortunately, the effect of volatile anaesthesia in combination with inhibition of fatty acid metabolism on ischaemic injury in T2DM hearts has not been studied yet, however, based on the use of these fatty acid inhibitors in models of T2DM it may be deduced that insulin resistance might be improved, thereby improving the impact of anaesthesia-induced cardioprotection.

#### Insulin

Glucose-insulin-potassium (GIK) infusion has been shown to reduce mortality in non-diabetic [100,101] and diabetic patients [102], and to reduce infarct size in rats [103]. However, also other results exist [104,105]. In the perioperative context, GIK infusion lowered glucose levels and other metabolic parameters [106] and improved perioperative outcomes, enhanced survival, decreased the incidence of ischaemic events [107] in T2DM patients during coronary artery bypass grafting (CABG).

The beneficial effects of GIK include increasing myocardial glucose uptake and glycogen content. It is suggested that insulin itself might be the major cardioprotective component. In isolated rat hearts, administration of insulin protected against ischaemia and reperfusion injury [108,109]. However, insulin treatment was not able to restore the lost cardioprotective capacity of sevoflurane in the type 1 diabetic heart [87].

Disadvantages of insulin infusion might be hypoglycaemia, which could be circumvented by additional glucose infusion (hyperinsulinaemic euglycaemic clamping). Insulin and dextrose infusion normalised postoperative whole body insulin sensitivity and substrate utilization in healthy patients during elective surgery [110]. During cardiac surgery, insulin and dextrose infusion maintained normoglycaemia in healthy [111] and T2DM [112] patients, however, hypolipidaemia was observed [113]. Further, it was shown in diabetic patients that isoflurane reduced postoperative markers of ischaemic injury after CABG, indicating a cardioprotective effect of isoflurane [114]. Preoperative treatment with glibenclamide prevented this protective effect, which was restored by changing glibenclamide preoperatively to insulin [114]. Taken together, these data suggest that perioperative glucose control by insulin may decrease the risk of postoperative mortality and morbidity.

#### Peroxisome proliferator-activated receptor agonists

Fibrates are selective peroxisome proliferator-activated receptor (PPAR)α agonists, which have lipid lowering effects, thereby improving insulin sensitivity. PPARα activation has been shown to reduce myocardial ischaemia and reperfusion injury in rat hearts [115,116]. Activation of PPARα in T2DM Goto-Kakizaki rat hearts reduced ischaemic injury [117], whereas in T2DM *db/db* mice PPARα activation did not affect the sensitivity to ischaemia and reperfusion even while myocardial glucose oxidation was increased and myocardial fatty acid oxidation reduced [47]. Moreover, sevoflurane reduced PPARα in whole blood compared to baseline [118], whereas during CABG sevoflurane reduced PPARα in right atrial tissue compared to propofol [11]. Based on these contrasting results, it might be interesting to study the effects of PPARα agonists combined with volatile anaesthesia.

Insulin-sensitizing drugs, such as thiazolidinediones have beneficial effects by activation of PPARγ. Rosiglitazone is the most selective PPARγ agonist and is widely used in the treatment of T2DM. PPARγ agonists have been shown to reduce myocardial ischaemia and reperfusion injury in rats [48,115,119,120]. Rosiglitazone has been shown to increase myocardial GLUT4 translocation [121] and glucose metabolism [122] in healthy and T2DM rat hearts. During myocardial ischaemia and reperfusion, it was shown that rosiglitazone treatment normalised ischaemic injury by improvement of the reduced glucose uptake in obese Zucker rats [44], and reduced ischaemic injury by improved myocardial insulin sensitivity and glucose oxidation in T2DM Zucker diabetic fatty rats [48], suggesting a role for PPARγ to influence myocardial substrate metabolism to optimise metabolic flexibility during myocardial ischaemia and reperfusion. Accordingly, it was shown that desflurane-induced cardioprotection during ischaemia and reperfusion was abolished by PPARγ inhibition in rabbits [123], suggesting a role for PPARγ in improvement of metabolic flexibility.

#### Metformin

Metformin, a biguanide with antihyperglycaemic properties, has been widely used in the treatment of obesity and T2DM and exerts its actions by enhancing insulin sensitivity. It is suggested that the glucose-lowering effects of metformin are mediated through the activation of AMPK, which has also been indicated to play an important protective role in the ischaemic mouse heart [124,125]. In non-diabetic rat hearts, metformin protects against ischaemic injury [126,127]. Accordingly, metformin provides cardioprotection against ischaemic injury in T2DM hearts from animals *in vivo*[125], but not *in vitro*[128]. The effects of volatile anaesthesia and metformin in ischaemic and T2DM hearts has not been studied yet. However, it has been shown that AMPK is involved in anaesthetic cardioprotection [41,129].

#### Glucagon-like peptide 1

Glucagon-like peptide 1 (GLP1) is a gut incretin hormone that is released in response to nutrient intake, stimulates insulin secretion and exerts insulinotropic and insulinomimetic properties. GLP1 has been shown to be protective in ischaemic rat hearts [130].

GLP1 has a short half-life of several minutes, due to rapid breakdown by dipeptidyl peptidase IV (DPP4). Exendin-4 is a peptide derived from the saliva of the gila monster which mimics GLP1, but is resistant to degradation by DPP4. Exenatide and liraglutide are synthetic GLP1 analogues, which mimic human GLP1 and are currently used for blood glucose-lowering therapy in T2DM. Exendin-4 [131], exenatide [132] and liraglutide [133] have been shown to reduce infarct size in animals, but also a neutral effect of liraglutide on myocardial infarct size was found [134]. Another possibility to circumvent the rapid breakdown of GLP1 is the use of a DPP4 inhibitor. However, inhibition of DPP4 by valine pyrrolidide in rats [130] or in DPP4 knockout mice [135] was not protective during myocardial infarction. It is suggested that the cardioprotective effect is a consequence of insulin, however, GLP1 has cardioprotective effects both *in vivo* and *in vitro*, whereby the latter is in absence of circulating insulin levels [130], suggesting a role for GLP1 in cardioprotection.

The mechanism behind the cardioprotective properties of GLP1 may, amongst others [136], rely on improving myocardial glucose metabolism. GLP1 increased glucose uptake in isolated mouse [137] and isolated healthy [138], hypertensive [139] and ischaemic/reperfused [138] rat hearts. Moreover, exenatide increased myocardial glucose uptake in healthy [140] and insulin resistant dilated cardiomyopathy [141] mice, whereas it did not alter myocardial glucose uptake in type 2 diabetic patients [142].

Exposure of healthy rats to isoflurane anaesthesia decreased GLP1 levels, without affecting DPP4 activity, insulin and glucose levels [143], suggesting impaired GLP1 secretion during isoflurane anaesthesia. However, the effect of volatile anaesthetics on GLP1 is scarcely studied and therefore no conclusion van be drawn.

Taken together, the above-discussed pharmacological interventions suggest that improving insulin sensitivity, and thereby improving myocardial flexibility, may be the most beneficial option in metabolically altered hearts in order to restore cardioprotective mechanisms. However, according to current clinical practice, oral hypoglycaemic agents are usually withheld before surgery in order to avoid associated adverse effects, such as perioperative hypoglycaemia or lactic acidosis. Therefore the (clinical) feasibility and safety of the proposed interventions should be carefully studied and weighted against the potential risk of these adverse effects.

### Preoperative health risk improvement

Based on 7 risk factors (physical inactivity, dietary pattern, obesity, smoking, high cholesterol, hypertension and elevated blood glucose levels), the 2020 impact goal of the American Heart Association is: “to improve the cardiovascular health by 20% while reducing deaths from cardiovascular diseases and stroke by 20%” [144]. Another possibility besides pharmacological intervention is preoperative lifestyle intervention, such as changing the dietary intake and stimulation of physical activity thereby losing weight and improving insulin sensitivity.

It has been shown by reducing dietary fat in rodents that diet-induced obesity is reversible [145-147]. In contrast, diet-induced obesity was not reversed by withdrawal of an energy dense diet [148]. Reversibility of diet-induced obesity is independent of the duration of the obese state [146], whereas long-term diet feeding did not reversed obesity [145]. Overall, these data suggest that changing dietary intake may have beneficial effects on health. However, there is only limited literature available that describes the effects of changing dietary balance on the heart.

In western diet-fed rats, lowering caloric intake improved systolic and diastolic function and prevented sevoflurane-induced cardiodepression (van den Brom *et al.*, unpublished observations). Accordingly, pacing-induced cardioprotection was lost by diet-induced hypercholesterolaemia, but restored after reversion to control diet [149], whereas caloric restriction by itself in healthy rats also has cardioprotective properties [150]. In conclusion, restriction of dietary fat seems an effective treatment to improve metabolic flexibility of the heart and thereby may be a possibility to reduce perioperative risk.

Obesity and T2DM are closely related to physical inactivity, and exercise could be a possible lifestyle intervention to reduce perioperative risk. The benefits of exercise with respect to obesity and T2DM are already recognized clinically [151]. However, the effects of exercise on myocardial infarction are contradictory. Exercise did not reduce myocardial ischaemic injury in rats [152], whereas others showed that exercise had protective effects in rat hearts [153-155]. The question remains if exercise has beneficial effects in obese and T2DM on myocardial function and ischaemia and reperfusion injury. Exercise was shown to reverse diet-induced obesity, insulin resistance and cardiomyocyte dysfunction [147], however, the effects of exercise on myocardial infarction in obese and T2DM with and without the effects of volatile anaesthesia is not known. Based on the above described results exercise might be a possible lifestyle intervention to reduce perioperative risk.

## Conclusions

Over the years, several mechanisms that are involved in anaesthesia-induced cardioprotection have been evaluated in the experimental setting. The existing evidence suggests that the obese and/or T2DM heart is less adaptable to cardioprotective interventions and that anaesthesia-induced cardioprotection is just a “healthy heart phenomenon”.

Differences between experimental models, the type of metabolic disease and the severity of myocardial substrate derangements challenge the identification of unifying mechanisms related to anaesthesia-induced cardioprotection in cases of obesity and T2DM. It might be deduced that interventional options should focus on recovery of the metabolic flexibility of the heart, especially by improving insulin sensitivity. Although changing lifestyle seems promising to reduce the susceptibility of the heart to intraoperative ischaemia and reperfusion injury, experimental data has not been translated into clinical data. Therefore more studies are required to elucidate whether these interventions have beneficial effects on perioperative outcome.

## Abbreviations

AMPK AMP: Activated Protein Kinase; ATP: Adenosine Triphosphate; CABG: Coronary Artery Bypass Grafting; CPT: Carnitine Palmitoyl Transferase; DCA: Dichloroacetate; DPP: Dipeptidyl Peptidase; FABPpm: Plasma Membrane Fatty Acid Binding Protein; FAT/CD36: Fatty Acid Translocase CD36; FATP: Fatty Acid Transport Protein; GIK: Glucose-Insulin-Potassium; GLP: Glucagon-Like Peptide; GLUT: Glucose Transporter; PPAR: Peroxisome Proliferator-Activated Receptor; T2DM: Type 2 Diabetes Mellitus.

## Competing interests

The authors declare that they have no competing interests.

## Authors’ contributions

CEvdB wrote the manuscript. CSEB, SAL and RAB reviewed the manuscript. CB wrote, reviewed and edited the manuscript. All authors read and approved the final manuscript.
